# *Elthusa winstoni* sp. n. (Isopoda, Cymothoidae), a new fish parasitic isopod from Hawaii

**DOI:** 10.3897/zookeys.661.11251

**Published:** 2017-03-15

**Authors:** Kerry A. Hadfield, Lillian J. Tuttle, Nico J. Smit

**Affiliations:** 1 Water Research Group, Unit for Environmental Sciences and Management, Potchefstroom Campus, North-West University, Private Bag X6001, Potchefstroom, 2520, South Africa; 2 Department of Biology, University of Hawai'i at Mānoa, Honolulu, Hawai'i 96822, USA

**Keywords:** Marine fish parasite, branchial cavity, Pacific Ocean, *Ctenochaetus strigosus*, *Acanthurus nigroris*

## Abstract

The new cymothoid species, *Elthusa winstoni* **sp. n.**, a branchial parasite of fishes from the family Acanthuridae Bonaparte, 1835 in Hawaii, is described and figured. The female adults can be distinguished by the strongly vaulted body and compacted body shape; rostrum with a small median point; short antennae which are close together (only 6 articles in both antennula and antenna); short and wide uropods extending to half the length of the pleotelson; short dactyli on pereopod 7; and large recurved robust setae on the maxilla. This is the first record of an *Elthusa* Schioedte & Meinert, 1884 species from the Hawaiian Islands and only the fifth cymothoid described from this region.

## Introduction

The cymothoid genus *Elthusa* Schioedte & Meinert, 1884 was first established in 1884, with *Elthusa emarginata* (Bleeker, 1857) the only known species at the time. Over a century later, Bruce (1990) revised the genus, reclassifying 21 species from *Livoneca* Leach, 1818 into *Elthusa*, and adding two new species. Since then, another four species have been described from New Caledonia and Mexico (Trilles and Justine 2004, 2006, 2010, Rocha-Ramírez et al. 2005). In their review of cymothoids, Smit et al. (2014) stated that there were 28 known *Elthusa* species. Since then, an *Elthusa* homonym has been corrected (Hadfield et al. 2016a), making a current total of 29 recognised species within the genus.

*Elthusa* has a global distribution (Rocha-Ramírez et al. 2005), being absent only from polar waters. While moderately well-known from the Caribbean, Australia and central Indo-Pacific, the vast central Pacific region has been scarcely studied, with recent records only from New Caledonia (Trilles and Justine 2004, 2006, 2010).

In Hawaii, Bunkley-Williams and Williams Jr (1996) listed the isopods known from Hawaiian fish hosts, recording only four cymothoid species, namely: *Anilocra gigantea* (Herklots, 1870); *Creniola breviceps* (Schioedte & Meinert, 1881); *Cymothoa recta* Dana, 1853; and *Ichthyoxenus puhi* (Bowman, 1960). This study is the first record of a gill-attaching *Elthusa* species in Hawaii, with both the male and female being described for the new species.

## Methods

During a research visit to the Smithsonian National Museum of Natural History in Washington DC (USA) in November 2014, an unnamed specimen originally collected in 1959 from *Acanthurus nigroris* Valenciennes, 1835, from Oahu, Hawaii, was observed. Recently, new material of the same species was collected from the gill rakers of a kole tang, *Ctenochaetus strigosus* (Bennett, 1828), speared by a SCUBA diver at approximately 20 m depth on 28 September 2015 in marine waters adjacent to the island of Niihau, Hawaii (22.00296, -160.11894). The host fish and parasites were stored frozen at-sea and initially examined approximately one month later at the Hawaii Institute of Marine Biology, Kaneohe, Hawaii. Isopods were subsequently removed from the gill chambers of the host fish and preserved in 70% ethanol. All specimens were processed following the techniques recorded in Hadfield et al. (2010, 2013). The female designated as the holotype (and male paratype) were minimally dissected in order to conserve the specimens. The species descriptions were prepared in DELTA (Descriptive Language for Taxonomy) using a general Cymothoidae character set (see Hadfield et al. 2016b). Isopod classification follows Brandt and Poore (2003) and host nomenclature follows that of FishBase (Froese and Pauly 2016) and *Catalog of Fishes* (Eschmeyer 2016).

**Abbreviations. AMNH** – American Museum of Natural History, New York, USA; **USNM** – National Museum of Natural History, Smithsonian Institution, Washington, D.C., USA; **TL** – total length; **W** – width.

## Taxonomy

### Suborder Cymothoida Wägele, 1989

#### Superfamily Cymothooidea Leach, 1814

##### Family Cymothoidae Leach, 1814

###### Genus Elthusa

Taxon classificationAnimaliaIsopodaCymothoidae

Schioedte & Meinert, 1884

Elthusa Schioedte & Meinert, 1884: 337.—Bruce 1990: 254.—Trilles 1994: 164.—Trilles and Randall 2011: 453–454.

####### Type species.

*Livoneca emarginata* Bleeker, 1857, by monotypy (Schioedte and Meinert 1884).

####### Remarks.

Diagnostic characters for *Elthusa* include a weakly vaulted body; pleonite 1 as wide as or slightly narrower than pleonite 2; posterior margin of cephalon not trilobed; wide pleon; antennula shorter than antenna, bases not in contact (varying from close together to wide apart); and pleopods all simple, lamellar. A revised diagnosis of the genus was provided by Bruce (1990) as well as Trilles and Randall (2011).

*Elthusa* can be regarded as one of the most morphologically varied cymothoid genera, with many *Elthusa* species still requiring detailed study and redescription. The antennula position (being close together or far apart), shape of the cephalon anterior margin, as well as the width of the pleon and pleonite 5 are some of the varying characters noted within species of this genus (Bruce 1990). This variation can cause confusion in genus identifications and may lead to incorrect species placement if not taken into account with all of the other genus characters.

###### Elthusa winstoni sp. n.

Taxon classificationAnimaliaORDOFAMILIA

http://zoobank.org/FDE6479B-D082-4699-9719-5BAB5181914E

**Figure 1.**
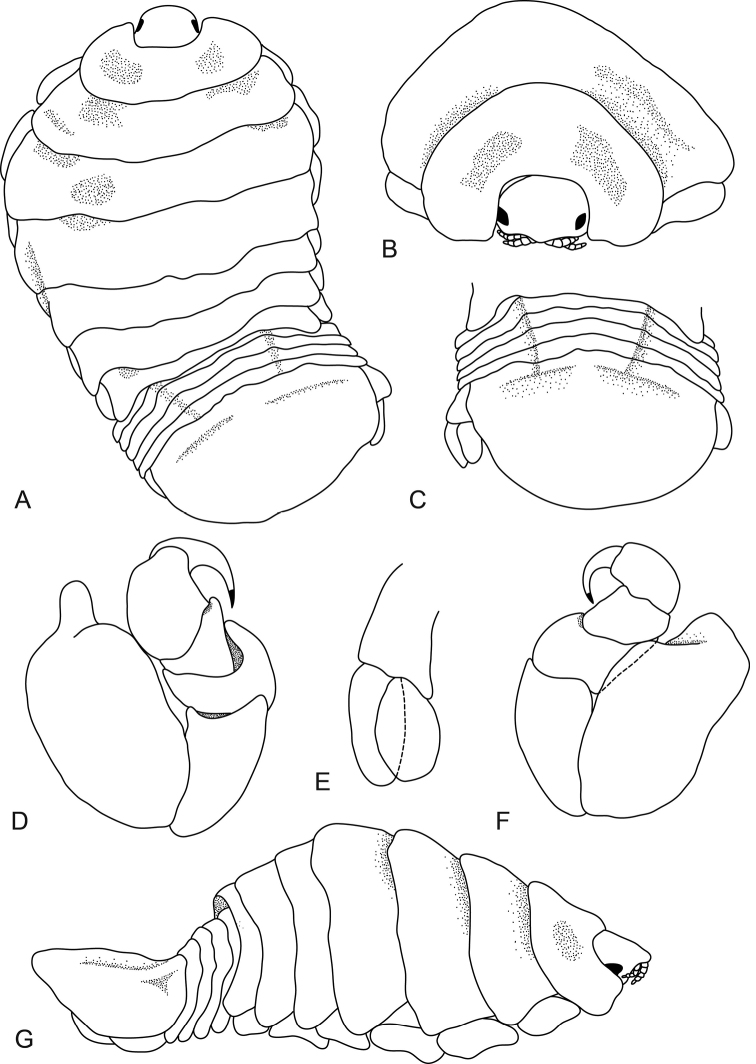
*Elthusa winstoni* sp. n., female holotype (17.5 mm) (AMNH_IZC 250217). **A** dorsal view **B** anterior view of pereonite 1, 2 and cephalon **C** dorsal view of pleotelson **D** pereopod 1 **E** uropod **F** pereopod 7 **G** lateral view.

**Figure 2.**
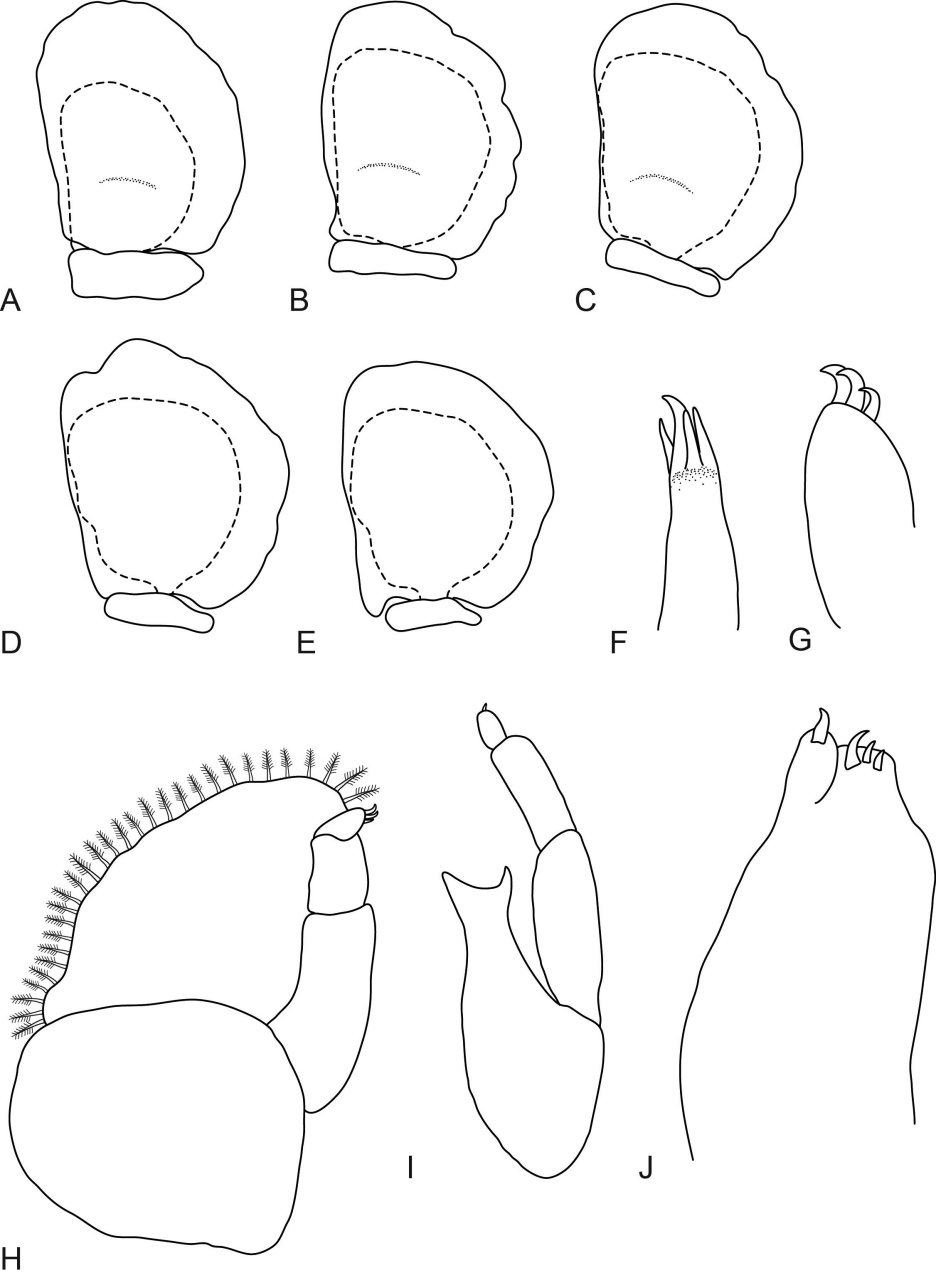
*Elthusa winstoni* sp. n., female holotype (17.5 mm) (AMNH_IZC 250217). **A–E** pleopods 1 to 5 respectively **F** tip of maxillule **G** tip of maxilliped article 3 **H** maxilliped with oostegite **I** mandible **J** maxilla.

**Figure 3.**
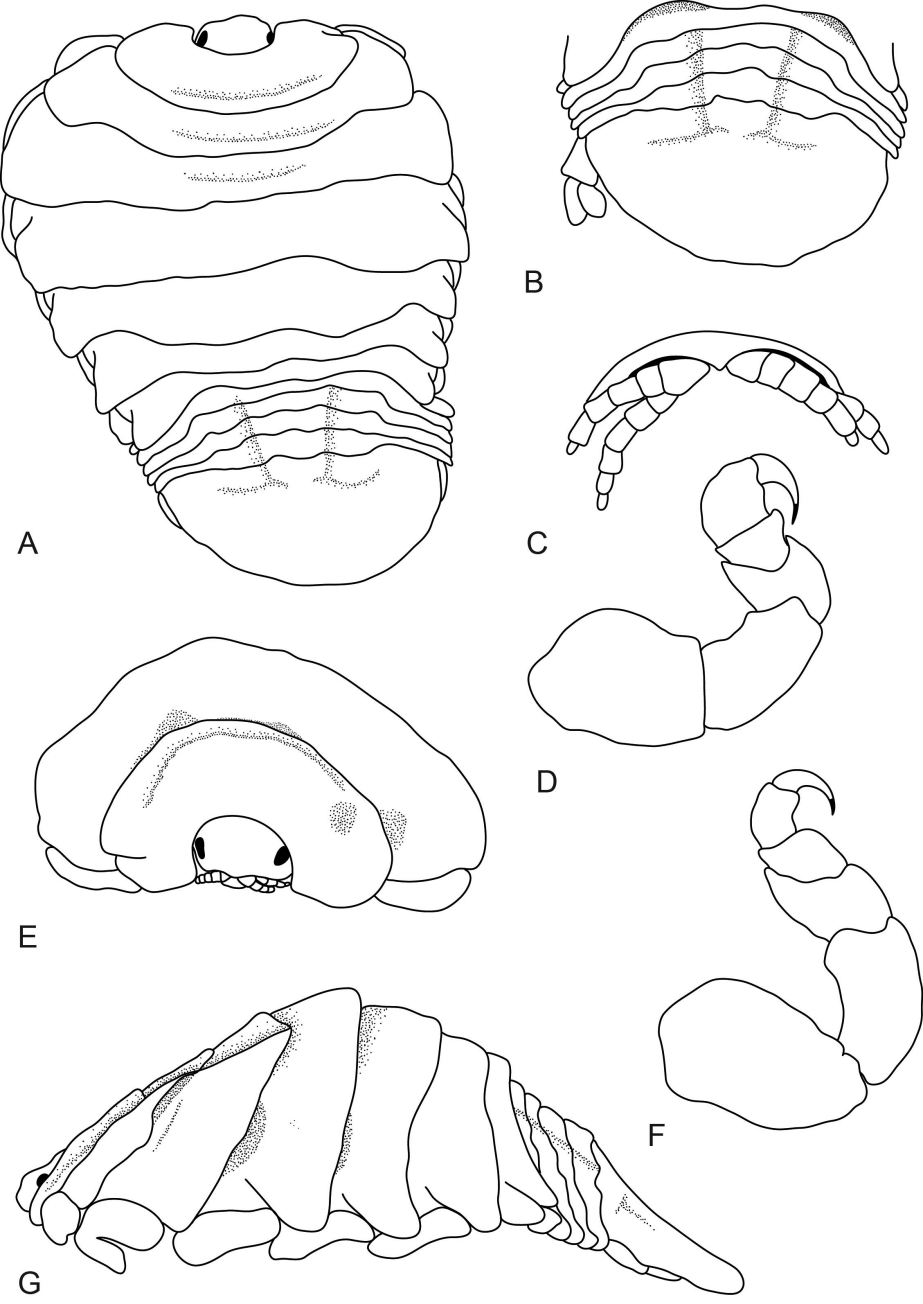
*Elthusa winstoni* sp. n., female (18 mm) (USNM 1256197). **A** dorsal view **B** dorsal view of pleotelson **C** ventral view of cephalon **D** pereopod 1 **E** anterior view of pereonite 1, 2 and cephalon **F** pereopod 7 **G** lateral view.

**Figure 4.**
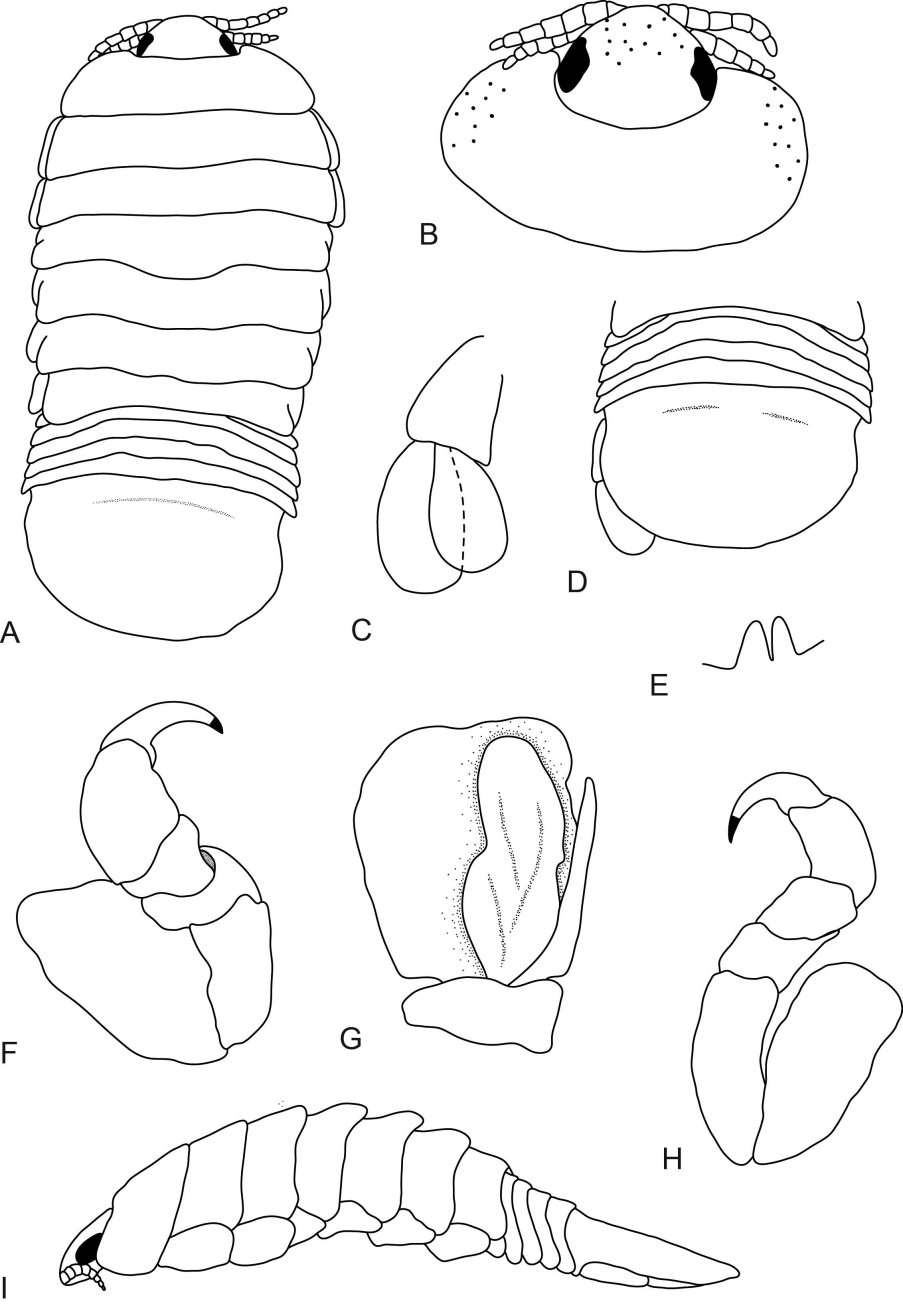
*Elthusa winstoni* sp. n., male paratype (8 mm) (AMNH_IZC 250218). **A** dorsal view **B** dorsal view of pereonite 1 and cephalon **C** uropod **D** dorsal view of pleotelson **E** penes **F** pereopod 1 **G** pleopod 2 with appendix masculina **H** pereopod 7 **I** lateral view.

Figures 1 , 2 , 3 , 4

####### Material examined.

*Holotype*. Female (17.5 mm TL; 11.5 mm W), partially dissected, from the gill rakers of kole tang, *Ctenochaetus strigosus* (Bennett, 1828), 18–21m deep, Niihau, Hawaii, 27.10.2015, col: Erik Franklin and Morgan Winston (AMNH_IZC 250217).

*Paratype*. Dissected male (8 mm TL; 3.5 mm W), male (8.5 mm TL; 4 mm W), same data as holotype (AMNH_IZC 250218).

####### Other material.

Ovigerous female (18 mm TL; 14 mm W), male (8 mm; 4 mm W), from the left gill cavity of the bluelined surgeonfish, *Acanthurus nigroris* Valenciennes, 1835, between Diamond Head and Koko Head, Oahu, Hawaii, 10.10.1959 (USNM 1256197).

####### Description.

*Holotype female.* Length 17.5 mm, width 11.5 mm.

*Body* compact, weakly twisted, 1.7 times as long as greatest width, wider anteriorly, dorsal surfaces rugose and strongly arched longitudinally, widest at pereonite 3, most narrow at pereonite 1, lateral margins slightly convex. *Cephalon* 0.7 times longer than wide, visible from dorsal view, square and deeply immersed in pereonite 1. *Frontal margin* forming rounded rostrum with small median point. *Eyes* oval with distinct margins, one eye 0.1 times width of cephalon; 0.25 times length of cephalon. *Pereonite 1* with slight indentations, anterior border straight, anterolateral angle with large broad projections, extend to anterior margin of eyes. Posterior margins of pereonites not smooth, with irregular nodules in certain areas. Coxae 2–3 wide, with posteroventral angles rounded; 4–7 rounded, not extending past pereonite margin. Pereonites 1–3 increasing in length and width; 4–7 decreasing in length and width; becoming more progressively rounded posteriorly. *Pleon* with pleonite 1 largely concealed by pereonite 7 and same width as other pleonites, partially visible in dorsal view; pleonites posterior margin not smooth. Distal ends of pleonite 2 partially overlapped by pereonite 7; posterolateral angles of pleonite 2 narrowly rounded. Pleonites 3–5 similar in form to pleonite 2. *Pleotelson* 0.6 times as long as anterior width, dorsal surface slightly depressed, lateral margins convex, posterior margin rounded.

*Antennula* approximately the same length as antenna, bases narrowly separated, consisting of 6 articles, extending to anterior margin of eye. *Antenna* consisting of 6 articles, extending to middle of the eye. *Mandibular molar process* ending in an acute incisor, with a single simple seta. *Maxillula* simple with 4 terminal robust setae. *Maxilla* mesial lobe partially fused to lateral lobe; lateral lobe with 3 recurved robust setae; mesial lobe with 1 large recurved robust seta. *Maxilliped* weakly segmented, with lamellar oostegite lobe, article 3 with 3 recurved robust setae.

*Pereopod 1* basis 1.5 times as long as greatest width; ischium 0.7 times as long as basis; merus proximal margin with bulbous protrusion; carpus with straight proximal margin; propodus as long as wide; dactylus slender, 0.9 times as long as propodus, 1.8 times as long as basal width. *Pereopod 7* same length as other pereopods, basis 1.8 times as long as greatest width; ischium 0.7 times as long as basis, without protrusions; merus proximal margin with slight bulbous protrusion, distal margin produced, 0.7 times as long as wide, 0.4 times as long as ischium; carpus 0.9 times as long as wide, 0.3 times as long as ischium, without bulbous protrusion; propodus 1.7 times as long as wide, 0.5 times as long as ischium; dactylus slender, 0.7 times as long as propodus, 2.4 times as long as basal width.

*Pleopods* without setae, simple. *Pleopod 1* exopod 1.3 times as long as wide, lateral margin strongly convex, distally broadly rounded, mesial margin straight; endopod 1.3 times as long as wide, lateral margin convex, distally broadly rounded, mesial margin straight; peduncle 2.8 times as wide as long, without retinaculae. Peduncle lobes absent. Pleopods 2–5 similar to pleopod 1.

*Uropod* half the length of pleotelson, peduncle 0.8 times longer than rami, peduncle lateral margin without setae. *Endopod* wide, apically rounded, 1.6 times as long as greatest width, lateral margin weakly convex, mesial margin weakly convex, terminating without setae. *Exopod* extending to end of endopod, 2.2 times as long as greatest width, apically rounded, lateral margin straight, mesial margin straight, terminating without setae.

*Male*. Length 8 mm, width 3.5 mm.

Male similar to female but much smaller. Body rectangular, body 2.1 times as long as wide. *Cephalon* with scattered chromatophores. *Pereonite 1* anterolateral margin broad with scattered chromatophores. *Antennula* bases separated, consisting of 8 articles, extending to posterior margin of eye. *Antenna* consisting of 8 articles, extending to posterior margin of cephalon. *Mandibular molar process* ending in an acute incisor. *Maxillula* simple with 4 terminal robust setae. *Maxilla* with 2 recurved robust setae. *Maxilliped* weakly segmented, with lamellar oostegite lobe, article 3 with 3 recurved robust setae. *Penes* opening flush with surface of sternite 7, tubercules separate, penial process 0.75 times as long as basal width. *Pleopod 2* appendix masculina with parallel margins, 0.8 times as long as endopod, distally narrowly rounded. *Uropods* same length as pleotelson.

####### Etymology.

Named after one of the collectors of the type specimen, Morgan Winston, who collected the type specimens while diving adjacent to the island of Niihau, Hawaii.

####### Distribution.

Known only from Hawaii.

####### Hosts.

Known only from the kole tang, *Ctenochaetus strigosus*, and the bluelined surgeonfish, *Acanthurus nigroris*, both from the family Acanthuridae Bonaparte, 1835.

####### Remarks.

*Elthusa winstoni* sp. n. can be distinguished from all congeners by the irregular, compact body shape; short antennae; short and wide uropods only extending to middle of the pleotelson; and a strongly vaulted body.

This species conforms with many of the *Elthusa* characters in having a wide pleon with pleonite 1 as wide a pleonite 2; the cephalon posterior margin is straight; the antennula bases are not in contact; and all of the pleopods are simple and lamellar. *Elthusa winstoni* sp. n. differs from other *Elthusa* species in the asymmetrical, strongly vaulted body and short antennae not extending past the cephalon (only 6 articles for both antennula and antenna in female, 8 in male). Furthermore, the mandibular palp is slender with only one seta, large recurved robust setae on maxilla, short dactyli on pereopod 7, and a very compact body (1.2 – 1.6 times as long as wide). There are no similar cymothoid species from Hawaii.

Many of the *Elthusa* hosts remain unknown; however, both *Acanthurus nigroris* and *Ctenochaetus strigosus* appear to be new hosts for *Elthusa* isopods. *Creniola breviceps* has previously been located on *Ctenochaetus strigosus* in Hawaii, but *Acanthurus nigroris* is a new host record for cymothoid isopods.

## Supplementary Material

XML Treatment for Elthusa

XML Treatment for Elthusa winstoni
